# Assessing the utility of Hsp90 gene for inferring evolutionary relationships within the ciliate subclass Hypotricha (Protista, Ciliophora)

**DOI:** 10.1186/s12862-020-01653-0

**Published:** 2020-07-16

**Authors:** Qi Zhang, Jiahui Xu, Alan Warren, Ran Yang, Zhuo Shen, Zhenzhen Yi

**Affiliations:** 1 Guangzhou Key Laboratory of Subtropical Biodiversity and Biomonitoring, School of Life Science, South China Normal University, Guangzhou, 510631 China; 2 Pilot National Laboratory for Marine Science and Technology (Qingdao), Qingdao, 266237 China; 3 Department of Life Sciences, Natural History Museum, London, SW7 5BD UK; 4 Institute of Microbial Ecology and Matter Cycle, School of Marine Sciences, Sun Yat-sen University, Zhuhai, 519000 China; 5 Southern Marine Science and Engineering Guangdong Laboratory (Zhuhai), Zhuhai, 519000 China

**Keywords:** Ciliates, Heat-shock protein 90 gene, Hypotricha, Phylogeny

## Abstract

**Background:**

Although phylogenomic analyses are increasingly used to reveal evolutionary relationships among ciliates, relatively few nuclear protein-coding gene markers have been tested for their suitability as candidates for inferring phylogenies within this group. In this study, we investigate the utility of the heat-shock protein 90 gene (Hsp90) as a marker for inferring phylogenetic relationships among hypotrich ciliates.

**Results:**

A total of 87 novel Hsp90 gene sequences of 10 hypotrich species were generated. Of these, 85 were distinct sequences. Phylogenetic analyses based on these data showed that: (1) the Hsp90 gene amino acid trees are comparable to the small subunit rDNA tree for recovering phylogenetic relationships at the rank of class, but lack sufficient phylogenetic signal for inferring evolutionary relationships at the genus level; (2) Hsp90 gene paralogs are recent and therefore unlikely to pose a significant problem for recovering hypotrich clades; (3) definitions of some hypotrich orders and families need to be revised as their monophylies are not supported by various gene markers; (4) The order Sporadotrichida is paraphyletic, but the monophyly of the “core” Urostylida is supported; (5) both the subfamily Oxytrichinae and the genus *Urosoma* seem to be non-monophyletic, but monophyly of *Urosoma* is not rejected by AU tests.

**Conclusions:**

Our results for the first time demonstrate that the Hsp90 gene is comparable to SSU rDNA for recovering phylogenetic relationships at the rank of class, and its paralogs are unlikely to pose a significant problem for recovering hypotrich clades. This study shows the value of careful gene marker selection for phylogenomic analyses of ciliates.

## Background

Evolutionary relationships of many ciliated protists (ciliates) remain unknown due to difficulties in species identification caused by their small size, complex morphological characters and high species diversity, although this topic is vital for understanding the evolution of life on Earth [1–4]. Most published molecular phylogenetic trees of ciliates are based on rDNA sequences, including the small subunit rDNA (SSU rDNA), large subunit rDNA (LSU rDNA) and the internal transcribed spacer (ITS) region [5–8]. All presently used markers give rise to artifacts in phylogenetic reconstructions resulting in the recovery of ambiguous relationships in gene trees [9, 10]. Analysis of sequences of multiple unlinked loci having different evolutionary histories, as opposed to linked SSU rDNA, LSU rDNA, and ITS regions, is necessary since phylogenetic bias might be common to loci that are linked [11]. Hence, protein-coding genes, such as alpha-tubulin, actin, Hsp70 and histone H4 genes, are increasingly used to reconstruct ciliate phylogenies [1, 3, 12–17]. With the development of phylogenomics, it is now possible to reconstruct phylogenetic relationships among ciliates based on analyses of hundreds of protein-coding genes [1, 18–22]. However, only a few of these protein-coding gene markers in comparatively few taxa have been tested for their suitability as candidates for inferring phylogenetic relationships among ciliates [23–25].

It has previously been shown that heat shock protein 90 (Hsp90) can resolve evolutionary relationships among eukaryotic taxa at high taxonomic rank, including alveolates and deep-branching dinoflagellates [26–29], and possibly also at genus level, e.g., *Paramecium* and *Tetrahymena* [30]. Furthermore, Hsp90 is ubiquitous and highly conserved in eukaryotic cells making it easy to amplify using PCR [31, 32]. As in other protein-coding genes used for molecular phylogenies, paralogs caused by gene duplication, especially recent ones, might be the biggest disadvantage of using Hsp90 in phylogenetic analyses [3, 33, 34]. Notably, gene duplications in ciliates are common due to extremely high copy numbers of genes caused by unique features such as nuclear dimorphism [6, 35, 36]. Hence, it is necessary to test whether Hsp90 gene paralogs will confound phylogenetic analyses before this gene is widely used for determining evolutionary relationships among ciliates.

The subclass Hypotricha is mainly defined by the patterns of ventral cirri arranged either in longitudinal files or in scattered groups and represents one of the most diverse groups within the Ciliophora [37]. This subclass has been the subject of many taxonomic revisions, particularly in the past three decades [1, 38–43]. Phylogenetic relationships within the Hypotricha are still poorly understood and the rapidly growing molecular phylogenetic studies have consistently questioned monophylies and assignments of many taxa [44–46].

In the present study, phylogenetic analyses based on the Hsp90 gene were carried out for 10 species (11 populations) of hypotrichs. The main aims were to examine whether Hsp90 is a suitable gene marker for resolving evolutionary relationships among ciliates using Hypotricha as an example, and to re-evaluate phylogenetic relationships within the subclass Hypotricha based on new Hsp90 sequence data.

## Results

### Hsp90 gene sequences of hypotrich ciliates

**Fig. 1:**
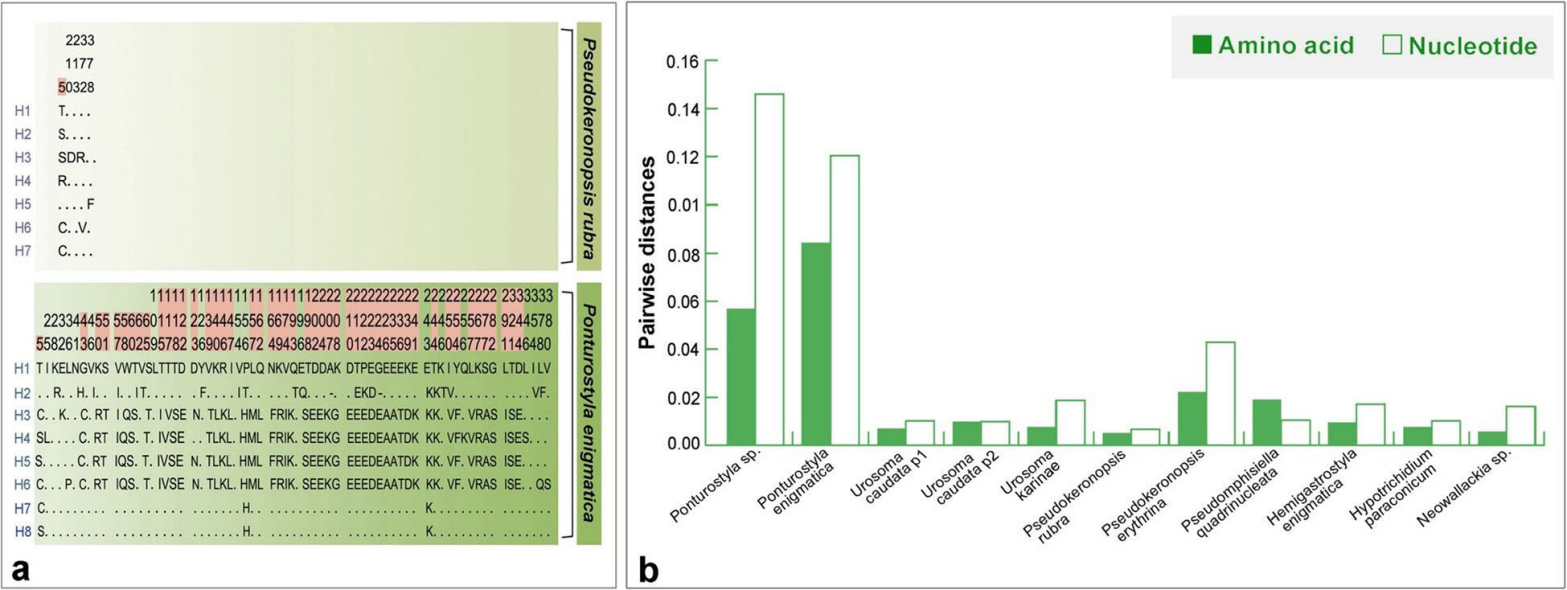
Amino acid variable sites and parsimony-informative sites in *Pseudokeronopsis rubra* and *Ponturostyla enigmatica* (**a**), and intra-population genetic distances of Hsp90 gene amino acid and nucleotide sequences (**b**). **a** Only variable sites are shown: parsimony-informative sites are shaded red; **b** genetic distances of amino acid and nucleotide are green solid and hollow boxes respectively

We obtained a total of 87 novel Hsp90 gene sequences from 11 populations of hypotrichs (Table 1). In order to avoid data redundancy we only used 85 distinct sequences in downstream analyses. The amplified fragment length of the Hsp90 gene was 1089–1119 base pairs (bp) with 41.2–60.1% GC content of which 543 sites (47.4%) were variable. Numbers of variable sites were highly divergent within each population, ranging from 16 (1.4%, *Pseudokeronopsis rubra*) to 216 (18.9%, *Ponturostyla enigmatica*). The third codon position exhibited the highest level of variation (42.0%). A total of 74 distinct amino acid sequences were detected among the 85 distinct nucleotide sequences. The 11 identical amino acid sequences were removed. The numbers of parsimony-informative and variable sites varied greatly at intra-population level. For instance, only one parsimony-informative site and five variable sites were present in *Pseudokeronopsis rubra*, while 48 and 67, respectively, were observed in *Ponturostyla enigmatica* (Fig. 1a).

**Table 1:** Comparison of Hsp90 nucleotide sequences (1145 bp) in 11 hypotrich populations

Populations	No. of distinct sequences	Total no. of variable sites (percentage of 1145 nucleotides)	No. of variable sites at first positions (percentage of variable sites)	No. of variable sites at second positions (percentage of variable sites)	No. of variable sites at third positions (percentage of variable sites)	Transversions (percentage of variable sites)	Transitions C/T (percentage of variable sites)	Transitions A/G (percentage of variable sites)
***Ponturostyla*sp.**	2 (2)	143 (12.5)	54 (37.8)	14 (9.8)	75 (52.4)	52 (36.4)	46 (32.2)	45 (31.4)
***Ponturostyla enigmatica***	6 (6)	216 (18.9)	71 (32.9)	38 (17.6)	107 (49.5)	159 (73.6)	26 (12.0)	31 (14.4)
***Urosoma caudata*p1**	15 (15)	33 (2.9)	5 (15.1)	9 (40.9)	19 (57.6)	25 (75.8)	4 (12.1)	4 (12.1)
***Urosoma caudata*p2**	5 (5)	22 (1.9)	7 (31.8)	6 (27.3)	9 (40.9)	16 (72.7)	2 (9.1)	4 (18.2)
***Urosoma caudata*(p1, p2)**	20 (20)	103 (9.0)	11 (10.7)	45 (43.7)	47 (45.6)	81 (78.6)	14 (13.6)	8 (7.8)
***Urosoma karinae***	10 (10)	44 (3.8)	5 (11.4)	11 (25.0)	28 (63.6)	29 (65.9)	9 (20.5)	6 (13.6)
***Pseudokeronopsis rubra***	9 (9)	16 (1.4)	4 (25.0)	4 (25.0)	8 (50.0)	12 (75.0)	2 (12.5)	2 (12.5)
***Pseudokeronopsis erythrina***	8 (8)	153 (13.4)	57 (37.3)	13 (8.5)	83 (54.2)	125 (81.7)	15 (9.8)	13 (8.5)
***Pseudoamphisiella quadrinucleata***	7 (7)	26 (2.3)	11 (42.3)	3 (11.5)	12 (46.2)	18 (69.2)	3 (11.5)	5 (19.3)
***Hemigastrostyla enigmatica***	7 (7)	38 (3.3)	7 (18.4)	9 (23.7)	22 (57.9)	24 (63.2)	9 (23.7)	5 (13.1)
***Hypotrichidium paraconicum***	9 (7)	23 (2.0)	5 (21.7)	8 (34.8)	10 (43.5)	16 (69.6)	3 (13.0)	4 (17.4)
***Neowallackia*sp.**	9 (9)	42 (3.7)	6 (14.3)	15 (35.7)	21 (50.0)	35 (83.3)	4 (9.5)	3 (7.2)
**Total**	87 (85)	543 (47.4)	208 (38.3)	107 (19.7)	228 (42.0)	440 (81.0)	56 (10.3)	47 (8.7)

Average nucleotide and amino acid pairwise distances within 11 hypotrich populations ranged from 0.0066 (*Pseudokeronopsis rubra*) to 0.1461 (*Ponturostyla* sp.), and 0.0051 (*Pseudokeronopsis rubra*) to 0.0843 (*Ponturostyla enigmatica*), respectively (Fig. 1b). Average inter-specific pairwise distances varied from 0.1062 (*Pseudokeronopsis erythrina* vs. *P. rubra*) to 0.3156 (*Hypotrichidium paraconicum* vs. *Neowallackia* sp.) for nucleotides, and 0.0424 (*Pseudokeronopsis erythrina* vs. *P. rubra*) to 0.1966 (*Pseudokeronopsis erythrina* vs. *Pseudoamphisiella quadrinucleata*) for amino acids (Table S1).

### Phylogenetic trees

**Fig. 2:**
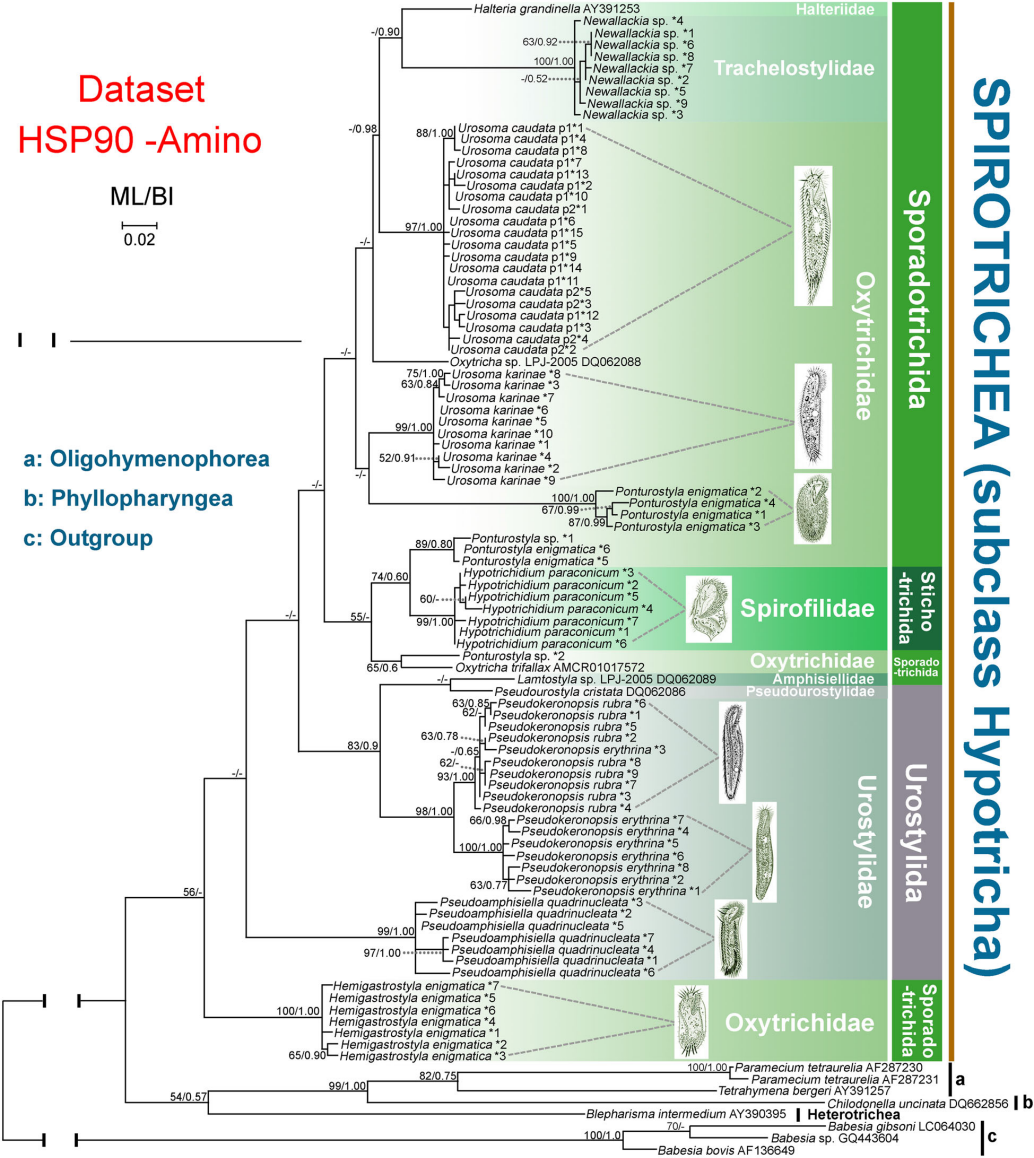
Phylogenetic tree constructed with HSP90-Amino dataset. Numbers near nodes are ML bootstrap support followed by BI posterior probability. Dashes (−) reflects bootstrap support values lower than 50 and disagreement between topologies. *N reflects numbering of distinct sequences. The scale bar corresponds to 2 substitutions per 100 nucleotide positions. Illustrations of species are original from previous investigations [44, 47–53]

In the Hsp90 amino acid (HSP90-Amino) tree, Oligohymenophorea and Spirotrichea were the only monophyletic classes with more than one representative species included in the analyses (Fig. 2). Another two classes, i.e. Heterotrichea and Phyllopharyngea, contain only one sequence each. Within Hypotricha, the orders Sporadotrichida and Urostylida were both monophyletic and order Stichotrichida was represented by only one species. Two out of six hypotrich families (Oxytrichidae and Urostylidae) represented by more than one species were polyphyletic. At the genus level, there was no sister relationship between the two representatives of *Urosoma*, i.e., *U. caudata* and *U. karinae,* and sequences of two *Ponturostyla* species fell into three different clades. In contrast, the two *Pseudokeronopsis* species clustered together in a strongly supported clade (98% ML, 1.00 BI). Only *U. caudata* was represented by more than one population (*U. caudata* p1 & p2), and their sequences formed a clade with reliable support (97% ML, 1.00 BI). For each of six populations, i.e., *Urosoma karinae* (99% ML, 1.00 BI), *Neowallackia* sp. (100% ML, 1.00 BI), *Pseudoamphisiella quadrinucleata* (99% ML, 1.00 BI), *Hypotrichidium paraconicum* (99% ML, 1.00 BI), *Hemigastrostyla enigmatica* (100% ML, 1.00 BI) and *Pseudokeronopsis rubra* (93% ML, 1.00 BI)*,* all sequences formed high to maximally supported clades. For five other populations, i.e., *U. caudata* p1, *U. caudata* p2, *Ponturostyla enigmatica*, *Ponturostyla* sp. and *Pseudokeronopsis erythrina,* this was not the case. For example, one sequence of *Pseudokeronopsis erythrina* grouped within the *P. rubra* clade. Within the class Oligohymenophorea, two sequences of *Paramecium tetraurelia* branched off with full support (100% ML, 1.00 BI) and clustered with *Tetrahymena bergeri* (AY391257) with moderate or low support (82% ML, 0.75 BI).

**Fig. 3:**
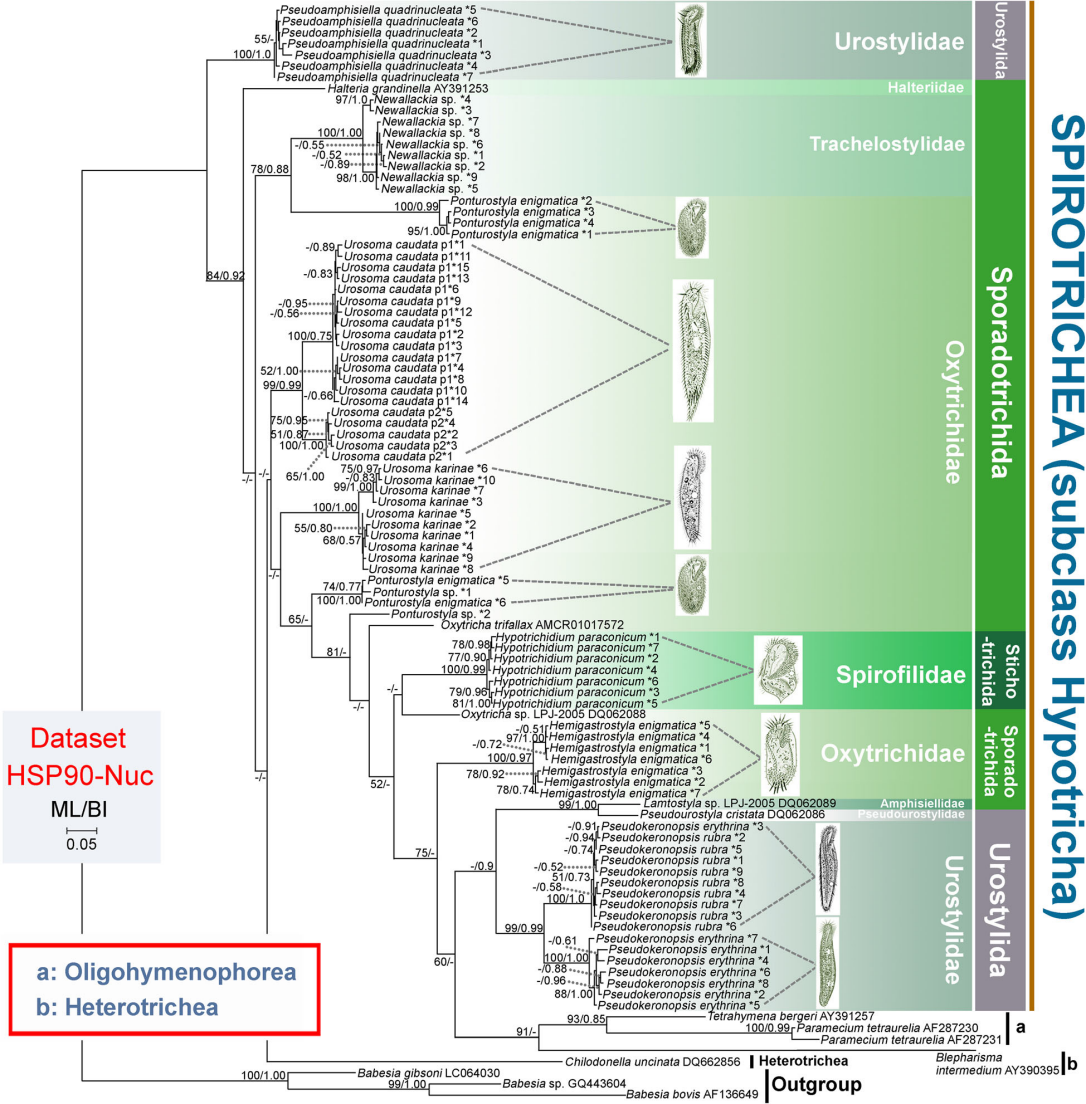
Phylogenetic tree constructed with HSP90-Nuc dataset. Numbers near nodes are ML bootstrap support followed by BI posterior probability. Dashes (−) reflects bootstrap support values lower than 50 and disagreement between topologies. *N reflects numbering of distinct sequences. The scale bar corresponds to 5 substitutions per 100 nucleotide positions. Illustrations of species are original from previous investigations [44, 47–53]

**Fig. 4:**
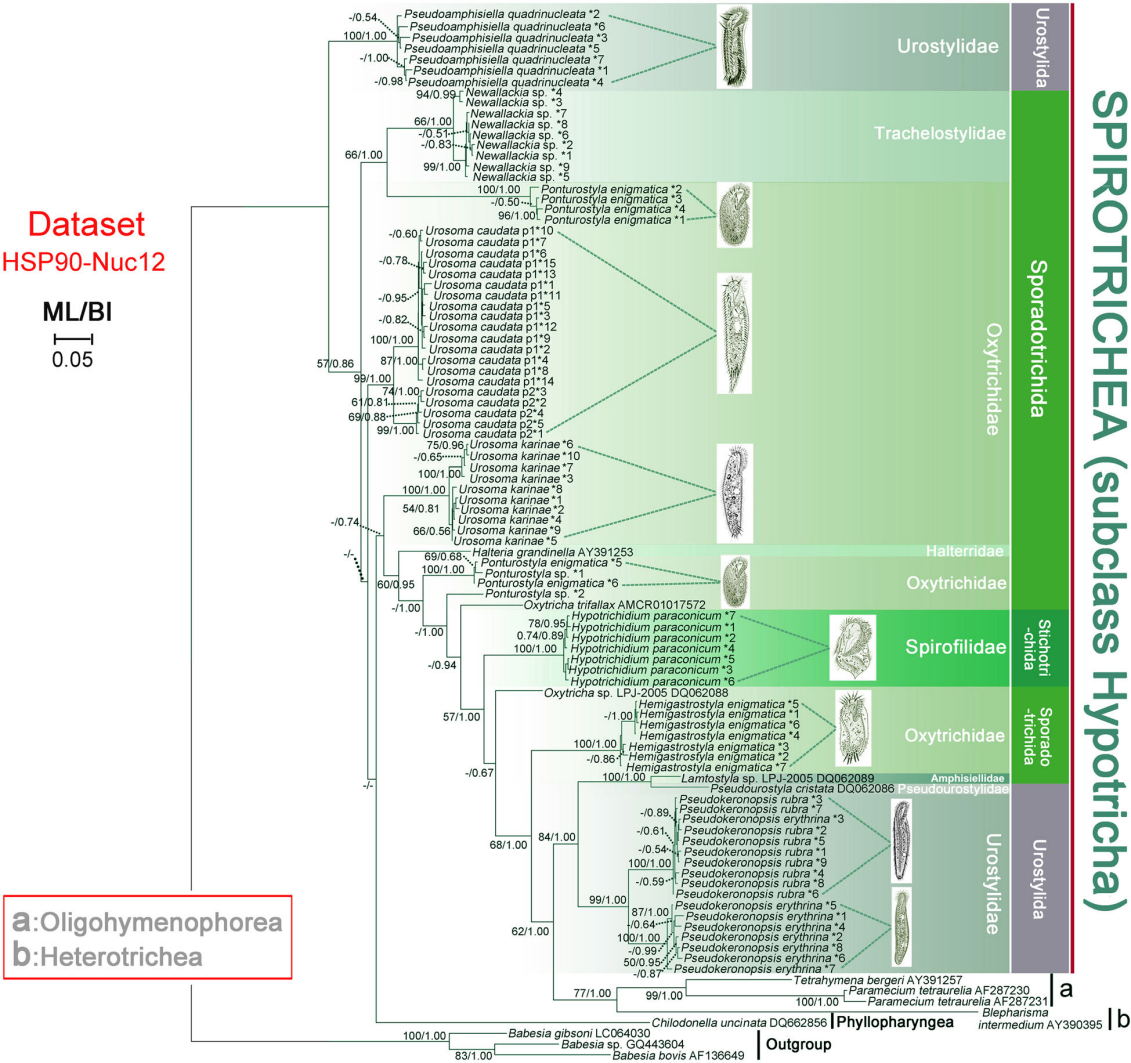
Phylogenetic tree constructed with HSP90-Nuc12 dataset. Numbers near nodes are ML bootstrap support followed by BI posterior probability. Dashes (−) reflects bootstrap support values lower than 50 and disagreement between topologies. *N reflects numbering of distinct sequences. The scale bar corresponds to 5 substitutions per 100 nucleotide positions. Illustrations of species are original from previous investigations [44, 47–53]

The topologies of the Hsp90 nucleotide (HSP90-Nuc) trees (Fig. 3) and Hsp90 nucleotide trees without third codon positions (HSP90-Nuc12) (Fig. 4) corresponded closely with that of the HSP90-Amino tree (Fig. 2), although there were three notable differences. Firstly, sequences of *Urosoma caudata* p1 and *U. caudata* p2 were separated from each other within a large clade and both populations appeared to be monophyletic in both the HSP90-Nuc and HSP90-Nuc12 trees, whereas they clustered together in the HSP90-Amino tree. Secondly, the clade comprising sequences of *Chilodonella uncinata* (DQ662856) occupied the basal position in the HSP90-Nuc and HSP90-Nuc12 trees, but it grouped with Oligohymenophorea and Heterotrichea in the HSP90-Amino tree. Thirdly, the subclass Hypotricha was polyphyletic in the HSP90-Nuc and HSP90-Nuc12 trees, but monophyletic in the HSP90-Amino tree.

**Fig. 5:**
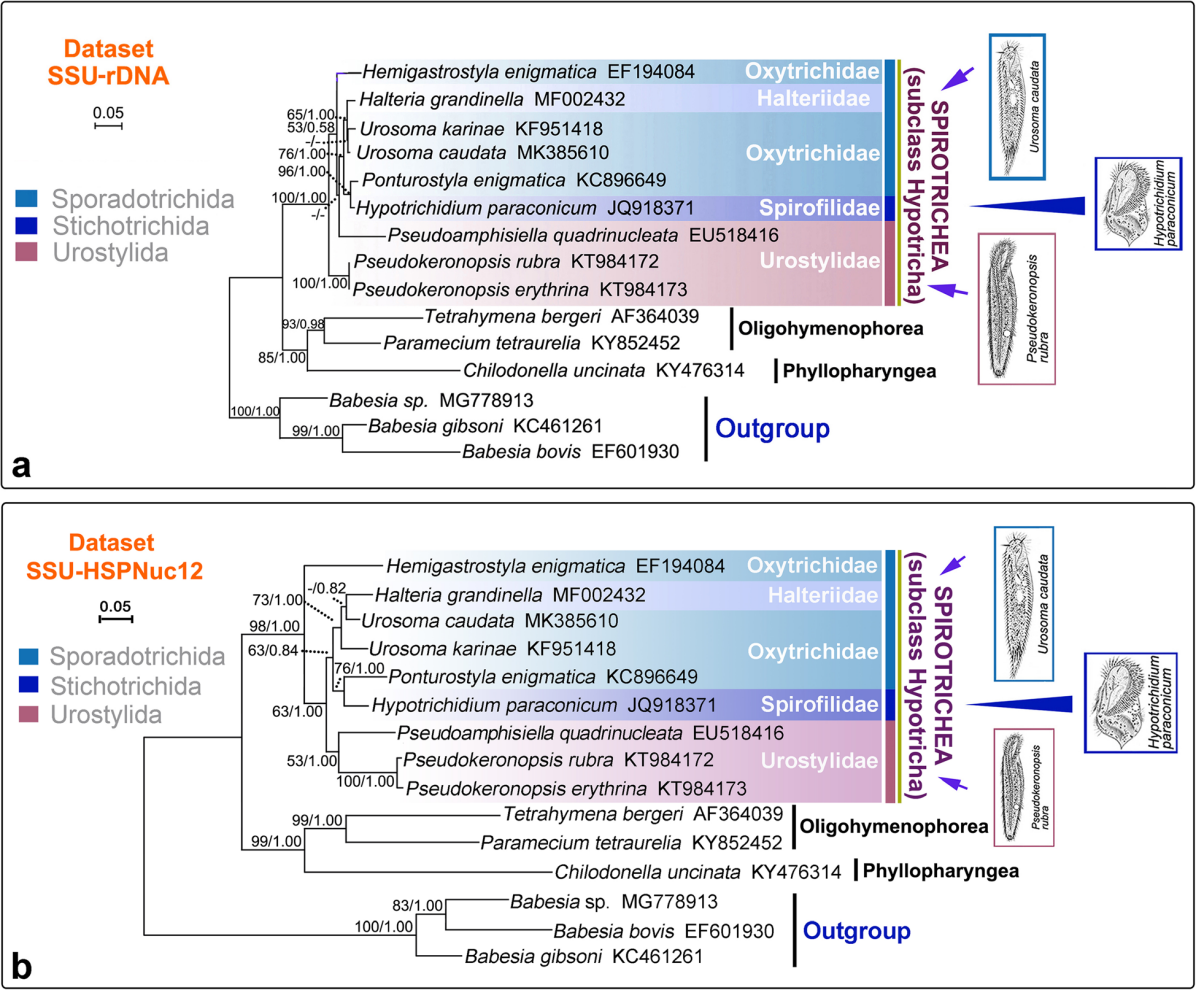
Phylogenetic trees constructed with SSU-rDNA dataset (**a**) and SSU-HSPNuc12 dataset (**b**). Numbers near nodes are ML bootstrap support followed by BI posterior probability. Dashes (−) reflects bootstrap support values lower than 50 and disagreement between topologies. The scale bar corresponds to 5 substitutions per 100 nucleotide positions. Illustrations of species are original from previous investigations [48–50]

The topology of the SSU-rDNA tree (Fig. 5a) was almost congruent with the HSP90-Amino tree (Fig. 2). The major difference was that the two *Urosoma* species clustered together in the SSU-rDNA tree, whereas they were separated into different clades in the HSP90-Amino tree.

The topology of the SSU-HSPNuc12 tree (Fig. 5b) was nearly identical to that of the SSU-rDNA tree (Fig. 5a) except that Urostylida/Urostylidae was monophyletic only in the SSU-HSPNuc12 tree (Fig. 5b). The monophyly of the subclass Hypotricha was also recovered in SSU-HSPNuc12 tree which is consistent with the HSP90-Amino and SSU-rDNA trees (Figs. 2 and 5a).

## Discussion

### Is Hsp90 gene a suitable gene marker for inferring hypotrich phylogeny?

Previous studies have shown that the best way to evaluate the utility of a gene marker is to compare its congruence with other data [9]. Some researchers have suggested that Hsp90 is as good or better than any other single gene marker for inferring eukaryote phylogeny [29]. Here, the reliability of the Hsp90 gene is compared with other gene markers, especially SSU rDNA, based on accepted monophyletic groups at different taxonomic ranks. In the present investigation the class Oligohymenophorea was monophyletic, which is consistent with previous reports based on a variety of gene markers [3, 6, 54, 55]. In contrast, the subclass Hypotricha was non-monophyletic in our HSP90-Nuc and HSP90-Nuc12 trees (Figs. 3 and 4) but monophyletic in the HSP90-Amino, SSU-rDNA and SSU-HSPNuc12 trees (Figs. 2 and 5). Non-monophyly in HSP90 trees (Figs. 3 and 4) might be caused by the rapid evolution of Hsp90 gene nucleotide sequences which is not suitable for reconstruction relationships of high-level taxa. It is suggested that amino acid sequences, rather than nucleotide sequences, of the Hsp90 gene are more suitable for the classification of ciliates. The subclass Hypotricha was monophyletic in most previous molecular phylogenetic trees based on a variety of gene markers [6, 56–59], although in a few studies it was non-monophyletic [1, 6, 33]. The utility of the Hsp90 gene is comparable to that of SSU rDNA at class level. Out of three hypotrich genera for which Hsp90 gene sequences from more than one species were available, two (*Urosoma* and *Ponturostyla*) were paraphyletic (Figs. 2 and 3). This suggests that the Hsp90 gene might lack sufficient phylogenetic signal for inferring evolutionary relationships at the genus level. Due to the lack of taxon coverage, we were unable to determine whether the Hsp90 gene yields sufficient phylogenetic signal to determine relationships at order or family levels. The order Sporadotrichida, and two families for which sequences from multiple species were available, were polyphyletic (Figs. 2, 3, 4, 5). This is consistent with previous findings based on SSU rDNA, actin and alpha-tubulin gene markers [43, 44, 46].

Multiple distinct Hsp90 nucleotide sequences were detected in every species, probably as a result of gene duplications. Of the 11 populations represented by more than one sequence, eight formed a well-supported clade in the HSP90-Nuc tree, the exceptions being *Pseudokeronopsis erythrina*, *Ponturostyla* sp. and *Ponturostyla enigmatica* (Fig. 3). This indicates that the Hsp90 gene duplications are recent and might have occurred after the separation of populations, suggesting that Hsp90 gene paralogs are unlikely to pose a substantial problem in defining hypotrich clades. Similarly, only recent duplication events have been detected in ciliates for the alpha-tubulin gene [17], this being the most widely used protein-coding gene for inferring evolutionary relationships in a range of ciliate groups [1, 13, 16, 17, 25]. Some studies, however, have concluded that the alpha-tubulin gene might not provide sufficient phylogenetic signal to resolve evolutionary relationships within some groups, e.g., *Spirostomum*, possibly due to the especially pronounced purifying selection [16, 60, 61]. In the case of the actin gene, the presence of ancient gene duplications and actin isoforms being too divergent means that it is not possible to resolve evolutionary relationships below the rank of class [3]. These findings indicate that gene duplication patterns of different protein-coding genes are variable depending on the gene and the ciliate group. More studies are therefore needed to test more genes and more ciliate groups.

### Phylogenetic relationships within the subclass Hypotricha

In the present study, each of the orders Sporadotrichida and Stichotrichida were paraphyletic and support values for some clades were low (Figs. 2, 3, 4, 5). This is consistent with previous molecular phylogenetic studies [3, 62–67]. It seems that gene markers with sufficient phylogenetic signal to recover the monophyletic Hypotricha are currently unavailable, although several (e.g., SSU rDNA, LSU rDNA, ITS and alpha-tubulin) have been widely used. This is reasonable considering that classification systems based on morphology, morphogenesis and gene sequences are not concordant with each other, probably due to the high diversity of ciliary and other somatic structures, as well as various modes of cortical development, within the Hypotricha [38–43, 68]. Currently, Hsp90 gene sequence data are available for only one species of Stichotrichida, namely *Hypotrichidium paraconicum*. Therefore, relationships within this order could not be analyzed. In contrast, several important phylogenetic relationships are recovered within the orders Sporadotrichida and Urostylida.

#### Sporadotrichida

It has been suggested that the family Oxytrichidae, which is characterized by typically having 18 front-ventral-transverse (FVT) cirri, should be divided into two subfamilies, the Oxytrichinae, with a flexible body, and the Stylonychinae, with a rigid body [38, 39]. All genera of Oxytrichidae included in the present study, i.e., *Oxytricha*, *Urosoma*, *Ponturostyla* and *Hemigastrostyla* belong to the subfamily Oxytrichinae. However, the subfamily Oxytrichinae was not monophyletic in any of the trees (Figs. 2, 3, 4, 5). This is consistent with previous studies based on SSU rDNA [69], actin [3] and alpha-tubulin genes [17], suggesting that the ontogenetic character which characterizes this group, viz. the participation of the posteriormost postoral ventral cirrus in primordia formation, might not be a synapomorphy. Furthermore, approximately Unbiased (AU) tests performed on HSP90-Nuc, HSP90-Nuc12, HSP90-Amino, SSU-rDNA and SSU-HSPNuc12 datasets rejected the monophyly of the subfamily Oxytrichinae (*P* < 0.05) (Table 2). The monophyly of the genus *Urosoma* was not supported in the HSP90-Nuc, HSP90-Nuc12, HSP90-Amino and SSU-HSPNuc12 trees, although this was not rejected by AU tests (*P* > 0.05, Table 2) (Figs. 2, 3, 4, 5b). Furthermore, no support values were revealed for the clade containing two *Urosoma* species shown in ML analyses based on the SSU-rDNA dataset (Fig. 5a). It is noteworthy that the monophyly of the genus *Urosoma* was not recovered in previous studies based on SSU rDNA sequences [47, 48]. The genus *Urosoma* might therefore be an artificial assemblage and its synapomorphies (i.e., frontoventral cirri arrange in a row with anterior cirrus (III/2) located slightly to the left, postoral ventral cirri in a dense cluster behind the buccal vertex, usually with two pretransverse ventral, five transverse cirri, one right and one left row of marginal cirri, and caudal cirri present) might be plesiomorphies of the subclass Hypotricha [38, 48]. Morphological, morphogenetic and molecular data for more taxa are required in order to resolve the systematics of Sporadotrichida.

**Table 2:** Approximately Unbiased (AU) test results of the monophyly of Oxytrichinae and *Urosoma* based on different datasets

Datasets	Topology constraints	-Ln^a^ Likelihood	AU value (*P*)
**HSP90-Nuc**	Oxytrichinae	18,926.77844542	< 0.001
	*Urosoma*	18,720.53438758	**0.693**
**HSP90-Nuc12**	Oxytrichinae	24,480.25572958	< 0.001
	*Urosoma*	24,253.92785203	**0.579**
**HSP90-Amino**	Oxytrichinae	6100.82366514	0.001
	*Urosoma*	6017.78810081	**0.576**
**SSU-rDNA**	Oxytrichinae	9661.66588815	0.001
**SSU-HSPNuc12**	Oxytrichinae	16,370.74872451	0.001
	*Urosoma*	16,280.08580004	**0.736**

^a^-Ln = negative value of natural logarithm; *P* values> 0.05 in bold

#### Urostylida

It is not surprising that *Pseudoamphisiella* was separated from the “core” Urostylida in most our trees (Figs. 2, 3, 4, 5a) since phylogenetic trees based on various gene markers have previously revealed the fragmentation of the order Urostylida into a “core” group and the rest [1, 46, 70, 71]. Considering that the monophyly of the “core” urostylid group was consistently recovered and other urostylid taxa usually had no robust assignment in our phylogenetic trees, we suggest that the morphological definition of the order Urostylida needs to be further refined.

## Conclusions

Our investigation indicates that, though lacking sufficient phylogenetic signal at the genus level, the Hsp90 gene is comparable to SSU rDNA for recovering evolutionary relationships at the rank of class and that its paralogs are unlikely to pose a significant problem for recovering hypotrich clades. The Hsp90 gene therefore has significant potential utility for determining the systematics of ciliates. The findings of this study also suggest that careful selection of nuclear protein-coding gene markers is needed for phylogenetic analyses of ciliates.

## Methods

### Culturing, DNA extraction, PCR amplification, and sequencing

All the ciliates used in this study are listed in Table 3. Pure cultures were maintained in Petri dishes at room temperature (approximately 25 °C) with rice grains to stimulate the growth of bacteria as food for the ciliates [72]. One or more cells of each culture were repeatedly washed in sterile water with the same salinity as that of the sampling site. Genomic DNA was extracted using REDExtract-NAmp Tissue PCR Kit (Sigma, St. Louis, MO, USA).

**Table 3:** Sampling information of 11 ciliate populations sequenced in this study

Organisms	Sampling locality Location	Sampling Salinity (‰)	GenBank No.	Morphological Data Sources
*Ponturostyla* sp*.*	Futian Mangrove (22°31′N; 114°04′E), Guangdong	20	MN892397 -MN892398	[53]
*Ponturostyla enigmatica*	Futian Mangrove (22°31′N; 114°04′E), Guangdong	5	MN892399 -MN892404	[53]
*Urosoma caudata* p1	Nansha Island (22°85′N; 113°51′E), Guangdong	25	MN892405 -MN892419	[48]
*Urosoma caudata* p2	Futian Mangrove (22°31′N; 114°04′E), Guangdong	8	MN892420 -MN892424	[48]
*Urosoma karinae*	Futian Mangrove (22°31′N; 114°04′E), Guangdong	7	MN892425 -MN892434	[2, 47]
*Pseudokeronopsis rubra*	Qingdao(36°03′N;120°20′E), Shandong	30	MN892435 -MN892443	[50]
*Pseudokeronopsis erythrina*	Pearl River Estuary (22°41′N; 113°38E), Guangdong	15	MN892444 -MN892451	[44]
*Pseudoamphisiella quadrinucleata*	Clear Water Bay (22°20′N, 114°17′E), Hong Kong	33.5	MN892452 -MN892458	[52]
*Hemigastrostyla enigmatica*	Daya Bay (22°43′N; 114°32′E), Guangdong	20	MN892459 -MN892465	[51]
*Hypotrichidium paraconicum*	Maipo Mangrove (22°29′N; 114°02′E), Hong Kong	18	MN892466 -MN892472	[49]
*Neowallackia* sp.	Futian Mangrove (22°31′N; 114°04′E), Guangdong	10	MN892473 -MN892481	[41]

PCR amplifications of the Hsp90 genes were performed using a TaKaRa Ex Taq DNA Polymerase Kit (TaKaRa Biomedicals, Japan). The primers used for Hsp90 gene amplification were Hsp90F4 (5′-CGGCACGTTCTACWSNAAYAARGA-3′) and Hsp90R3 (5′-GGTCTTTCTTCTGGCGTGTTCAGTGTA-3′) [26]. PCR conditions were: 2 min initial denaturation (95 °C), followed by 35 cycles of 45 s at 92 °C, 45 s at 48 °C and 1.5 min at 72 °C, with a final extension of 10 min (72 °C). Reactions were run in a total volume of 50 ul containing 5 ul 10 × Ex Taq DNA Polymerase buffer, 5 ul 2.5 mmol/L dNTP mix, 0.4 ul Ex Taq DNA Polymerase, 0.8 ul of each primer (25 mM), 2 ul of template DNA, and 36 ul of autoclaved double-distilled water.

After confirmation of the amplified DNA by 1.0% agarose gel, a single bright band containing the target DNA was purified using the Universal DNA purification kit (TIANGEN, Beijing, China). Subsequently, the purified PCR products were cloned with the pMD18-T Cloning Vector (TaKaRa Biomedicals, Japan) and DH5-α *E. coli* cells. A total of 2–15 positive colonies from each population were sequenced in both directions in an ABI Prism 377 Automated DNA Sequencer (Majorbio sequencing facility, Shanghai, China) using primers M13-F47 and M13-R48.

### Sequence analyses and construction of phylogenetic trees

Five datasets were included in the phylogenetic analyses: (1) HSP90-Nuc (98 sequences in total, i.e., Hsp90 nucleotide sequences including 85 that were distinct and newly obtained from the present study together with all 10 ciliate sequences available from the GenBank database); (2) HSP90-Amino (98 sequences in total, i.e., corresponding amino acid sequences of HSP90-Nuc); (3) HSP90-Nuc12 (98 sequences in total, i.e. HSP90-Nuc including only first two codon positions); (4) SSU-rDNA (15 sequences in total, i.e., corresponding SSU rDNA sequences of species in Dataset HSP90-Nuc, expect for three that were absent in GenBank, namely *Oxytricha trifallax*, *Newallackia* sp. and *Blepharisma intermedium*); (5) SSU-HSPNuc12 (15 sequences in total: two-gene combined dataset including SSU-rDNA and HSP90-Nuc including only first two codon positions). In each dataset, three species of Apicomplexa were chosen as the outgroup.

The Hsp90 nucleotide sequences were translated into amino acid sequences by DAMBE 6.3.0.1 software [73]. Intra- and inter-population genetic distances were calculated using MEGA 7.0 [74]. Mean pairwise nucleotide distances were calculated using the Kimura 2-parameter correction model [75]. Mean pairwise amino acid distances were calculated using the Poisson model [76]. Multiple sequence alignments of all nucleotide and amino acid sequences were performed with CLUSTAL W implemented in BioEdit v.7.0.1 [77] and then manually modified in order to trim both ends. Alignments used for subsequent phylogenetic analyses included the following numbers of positions: 1743 (HSP90-Nuc), 386 (HSP90-Amino), 1162 (HSP90-Nuc12), 1806 (SSU-rDNA), and 2968 (SSU-HSPNuc12). Models for nucleotide datasets were selected under the Akaike information criterion (AIC) by MrModeltest [78]. GTR + I + G was the best-fitted model for datasets HSP90-Nuc and SSU-rDNA. GTR + G was the best-fitted model for datasets HSPNuc12, SSU-HSPNuc12, as well as first and second codon positions of Hsp90 nucleotide sequences. Blosum62 + I + G was the best-fitted model for the amino acid dataset (HSP90-Amino) selected by AIC as implemented in ProtTest 1.4 [79]. Bayesian inference (BI) analyses were performed with MrBayes 3.1.2 [80]. Markov chain Monte Carlo (MCMC) simulations were run with two sets of four chains for 1,000,000 generations with trees sampled every 100 generations. The first 2500 trees were discarded as burn-in. The remaining trees were retained to generate a consensus tree and to calculate the posterior probabilities (PP) of all branches using a majority-rule consensus approach. A maximum likelihood tree (ML) was constructed using online software RAxML-HPC2 on XSEDE (http://www.phylo.org/). The branches of the resulting tree were evaluated by the GTRGAMMA model of nucleotide substitution and the PROTGAMMA model of amino substitution. MEGA7.0 [74] was used to visualize tree topologies. Terminology and systematic classification follow Gao et al., (2016) [1] and Lynn (2008) [43].

Constrained ML trees of Oxytrichinae species and *Urosoma* species were generated based on HSP90-Nuc, HSP90-Nuc12, HSP90-Amino, SSU-rDNA and SSU-HSPNuc12 datasets by RAxML; the remaining taxa were unspecified. Resulting constrained topologies and the unconstrained ML topologies were calculated using PAUP* v.4.0 [81] and were analyzed with AU test [82] in CONSEL v0.1j [83].

## Supplementary information

**Additional file 1: Supplementary Table S1.** Inter-specific genetic distances of Hsp90 nucleotide and amino acid sequences.

## Data Availability

Sequence data are available in GenBank (Accession Numbers: MN892397-MN892481). The datasets used and/or analyses during the current study are available from the corresponding author Zhenzhen Yi on reasonable request.

## Abbreviations

AIC: Akaike information criterion
BI: Bayesian inference
DNA: Deoxyribonucleic acid
GTR + I + G: General time reversible + invariable sites + gamma model of nucleotide substitution
Hsp/HSP: Heat-shock protein gene
HSP90-Amino: Hsp90 amino acid sequences dataset
HSP90-Nuc: Hsp90 nucleotide sequence datasets
HSP90-Nuc12: Hsp90 nucleotide sequences dataset without third codon positions
ITS: Internal transcribed spacer
LSU rDNA: Large subunit rDNA
ML: Maximum likelihood
p1: Population 1
p2: Population 2
PCR: Polymerase chain reaction
sp.: Species
SSU rDNA: Small subunit rDNA
SSU-rDNA: Small subunit rDNA sequence dataset
SSU-HSPNuc12: Two-gene combined dataset including SSU-rDNA and HSP90-Nuc12
