# Supplementary material for: Assessing the utility of Hsp90 gene for inferring evolutionary relationships within the ciliate subclass Hypotricha (Protista, Ciliophora)
Source: BMC Evol Biol. 2020 Jul 16;20:86. doi: 10.1186/s12862-020-01653-0 (PMC7364784; doi:10.1186/s12862-020-01653-0)
Supplement: Supplementary file 1 — Additional file 1: Supplementary Table S1. Inter-specific genetic distances of Hsp90 nucleotide and amino acid sequences. [file 12862_2020_1653_MOESM1_ESM.docx]

**Supplementary Table S1**

Inter-specific genetic distances of Hsp90 nucleotide and amino acid sequences.

| **Species** |  | **1** | **2** | **3** | **4** | **5** | **6** | **7** | **8** | **9** | **10** |
| --- | --- | --- | --- | --- | --- | --- | --- | --- | --- | --- | --- |
| **1. *Ponturostyla* sp.** |  | - | 0.1146 | 0.1089 | 0.0922 | 0.1269 | 0.1410 | 0.1497 | 0.1220 | 0.0605 | 0.1497 |
| **2. *Ponturostyla enigmatica*** |  | 0.1817 | - | 0.1397 | 0.1270 | 0.1474 | 0.1625 | 0.1826 | 0.1475 | 0.1177 | 0.1755 |
| **3. *Urosoma caudata*** |  | 0.1636 | 0.2050 | - | 0.0794 | 0.1346 | 0.1521 | 0.1533 | 0.1360 | 0.1128 | 0.1228 |
| **4. *Urosoma karinae*** |  | 0.1729 | 0.2253 | 0.1553 | - | 0.1395 | 0.1511 | 0.1503 | 0.1231 | 0.0901 | 0.1196 |
| **5. *Pseudokeronopsis rubra*** |  | 0.2262 | 0.2680 | 0.2426 | 0.2239 | - | 0.0424 | 0.1894 | 0.1279 | 0.1294 | 0.1504 |
| **6. *Pseudokeronopsis erythrina*** |  | 0.2643 | 0.3001 | 0.2804 | 0.2544 | 0.1062 | - | 0.1966 | 0.1374 | 0.1494 | 0.1657 |
| **7. *Pseudoamphisiella quadrinucleata*** |  | 0.2062 | 0.2343 | 0.1767 | 0.2211 | 0.2586 | 0.2913 | - | 0.1708 | 0.1524 | 0.1881 |
| **8. *Hemigastrostyla enigmatica*** |  | 0.2251 | 0.2776 | 0.2542 | 0.2581 | 0.2196 | 0.2208 | 0.2643 | - | 0.1338 | 0.1527 |
| **9. *Hypotrichidium paraconicum*** |  | 0.1912 | 0.2717 | 0.2506 | 0.2319 | 0.2413 | 0.2496 | 0.2890 | 0.2334 | - | 0.1510 |
| **10. *Neowallackia* sp.** |  | 0.2030 | 0.2026 | 0.1760 | 0.2034 | 0.2663 | 0.3129 | 0.2247 | 0.2892 | 0.3156 | - |

Note: Nucleotide distances are shown below the diagonal and amino acid distances are shown above the diagonal. Bold numbers correspond to the 10 species named in the first column.
